# BioTM Buzz (Volume 3, Issue 2)

**DOI:** 10.1002/btm2.10091

**Published:** 2018-05-21

**Authors:** Aaron C. Anselmo

**Affiliations:** ^1^ Division of Pharmacoengineering and Molecular Pharmaceutics, Eshelman School of Pharmacy, University of North Carolina at Chapel Hill Chapel Hill, NC 27599

## Volume 3, Issue 2

### MULTIPLEXED SIRNA DELIVERY

There is an urgent need to develop life‐saving therapies for diseases such as mantle cell lymphoma that otherwise have no cure. In this issue of Bioengineering & Translational Medicine, engineers from Carnegie Mellon University describe a novel RNAi‐based approach to target mantle cell lymphoma at the mRNA level to prevent cell proliferation. The authors used lipid nanoparticles to deliver an siRNA cocktail to mantle cell lymphoma cells that silenced three genes associated with cell proliferation. Three days post‐treatment with the siRNA cocktail, almost 75% of JeKo‐1 mantle cell lymphoma cells underwent apoptosis. Furthermore, lymphoma cells treated with the multiplexed siRNA lipid nanoparticles exhibited an 80% growth reduction compared to siRNA‐free lipid nanoparticle controls. Importantly, the addition of each siRNA to the cocktail increased cancer cell death and decreased proliferation, even when the total siRNA dose remained constant. In the case of cancer, one of the main advantages of RNAi‐based therapy is that it is mechanistically distinct compared to commonly used treatment modalities (e.g. chemotherapy, immunotherapy). As such, RNAi therapy has the potential to work synergistically with current treatment options, which may enable dose reduction of chemotherapy or provide additional options to the patient upon relapse.

DOI: 10.1002/btm2.10088

### LEAVING A PIECE BEHIND

A number of nanoparticle‐based therapeutics have been approved for use in the clinic to treat a variety of diseases. Recently, next‐generation nanoparticle technologies, such as antibody‐targeted nanoparticles, have entered clinical trials. Highlighted in this issue of *Bioengineering & Translational Medicine*, researchers from the University of California, Santa Barbara, the CFD Research Corporation, and the John A. Paulson School of Engineering and Applied Sciences and the Wyss Institute at Harvard University describe how next‐generation nanoparticle systems may require additional translational considerations. This work demonstrated that surface‐functionalized nanoparticles undergo a loss of surface molecules in response to flow forces that occur under physiological vasculature conditions. The surface of camptothecin nanoparticles were functionalized with polyethylene glycol (PEG) or PEG‐folic acid. Dominating factors that influence the extent of surface molecule loss include: (a) the method of attachment, where physically adsorbed PEG exhibited increased loss as compared to conjugated PEG in the presence of endothelial cells, and (b) the addition of folic acid onto the PEG‐nanoparticle surface, where the highest loss of surface molecules was observed, perhaps due to folic acid/endothelial cell interactions. The importance of this work is twofold as it highlights the need for additional considerations of next‐generation nanoparticle therapeutics and also motivates the use of microfluidic devices and more complex organs‐on‐chips to evaluate nanoparticle‐cell interactions under physiological conditions that are not possible to recreate in widely used static systems.

DOI: 10.1002/btm2.10089

## Recent literature

### SAMPLING HARD TO REACH FLUIDS

Recent work from the Mark Prausnitz lab in the School of Chemical and Biomolecular Engineering at the Georgia Institute of Technology describes a topical microneedle patch that can puncture skin and facilitate the collection of interstitial fluid. This technology could be used for minimally invasive diagnosis of diseases such as skin cancer or for monitoring concentration of delivered drugs. This work leveraged experimental approaches in porcine skin which determined that best microneedle technology, as determined by the volume of interstitial fluid extracted from skin, utilized a pressure‐driven convection mechanism (diffusion, capillary flow, osmotically driven flow mechanisms were also tested). Supporting these results, theoretical approaches elucidated that the rate‐limiting step in the transport of interstitial fluid occurred in the dermis and thus convective‐based approaches should enable increased collection of interstitial fluid as compared to diffusion‐based approaches. These findings enabled optimization of the microneedle patch which was capable of collecting over 1 μL of interstitial fluid from human subjects in 20 min. These human interstitial fluid samples were then tested for glucose and total protein content, which were quantitatively comparable with results from a capillary blood stick. This work is an example of how a rationally designed technology, supported by *in vitro* experimental approaches and theoretical methods, can be translated into the clinic.

*Samant and Prausnitz, Proceedings of the National Academy of Sciences. 2018; doi*: 10.1073/pnas.1716772115

